# Human-Induced Pluripotent Stem Cells Manufactured Using a Current Good Manufacturing Practice-Compliant Process Differentiate Into Clinically Relevant Cells From Three Germ Layers

**DOI:** 10.3389/fmed.2018.00069

**Published:** 2018-03-15

**Authors:** Mehdi Shafa, Fan Yang, Thomas Fellner, Mahendra S. Rao, Behnam Ahmadian Baghbaderani

**Affiliations:** ^1^Lonza Walkersville, Inc., Walkersville, MD, United States; ^2^NxCell Inc, Novato, CA, United States; ^3^Q Therapeutics, Salt Lake City, UT, United States

**Keywords:** current good manufacturing practices, induced pluripotent stem cells, differentiation, cell therapy, regenerative medicine

## Abstract

The discovery of reprogramming and generation of human-induced pluripotent stem cells (iPSCs) has revolutionized the field of regenerative medicine and opened new opportunities in cell replacement therapies. While generation of iPSCs represents a significant breakthrough, the clinical relevance of iPSCs for cell-based therapies requires generation of high-quality specialized cells through robust and reproducible directed differentiation protocols. We have recently reported manufacturing of human iPSC master cell banks (MCB) under current good manufacturing practices (cGMPs). Here, we describe the clinical potential of human iPSCs generated using this cGMP-compliant process by differentiating them into the cells from all three embryonic germ layers including ectoderm, endoderm, and mesoderm. Most importantly, we have shown that our iPSC manufacturing process and cell culture system is not biased toward a specific lineage. Following controlled induction into a specific differentiation lineage, specialized cells with morphological and cellular characteristics of neural stem cells, definitive endoderm, and cardiomyocytes were developed. We believe that these cGMP-compliant iPSCs have the potential to make various clinically relevant products suitable for cell therapy applications.

## Introduction

Recent advances in the field of pluripotent stem cells (PSCs) and establishing specialized PSC derivatives have made a remarkable impact in understanding the biology of these cells and offered tremendous potential in personalized medicine for the treatment of degenerative diseases. In particular, the remarkable discovery of somatic cell reprogramming by Shinya Yamanka (1) has dramatically impacted the field of drug discovery, toxicity testing, in-a-dish disease modeling, gene therapy, and gene editing during the last decade (2, 3). This is mostly due to enormous capability of human induced pluripotent stem cells (iPSCs) to expand *in vitro* and their inherent potential to differentiate into any cell type in the body, making them a precious source for clinical purposes (4). On the other hand, the increasing incidence of degenerative disorders, inefficiency of existing treatments, and the scarcity of functional primary human somatic cells are significantly increasing the demand for stem cell-based therapeutic approaches.

Patient-derived iPSCs have been used to model several human genetic diseases and to successfully produce clinically relevant differentiated cells that display disease pathogenesis (5–8). Furthermore, recent progresses in the development of directed differentiation protocols using human iPSCs into various cell types (9–11) have already resulted in the start of early autologous clinical trials (12). However, establishment of a robust directed differentiated procedure starting from high-quality cells manufactured using a robust and current good manufacturing practice (cGMP)-compliant process still remain a major challenge in enabling clinical utility of iPSC-based therapies. In particular, inherent difficulties in achieving high-quality cGMP grade PSCs and their progenies is a major obstacle in cell-based therapy and should be overcome before these cell types can be used to treat diseases (13).

We have recently reported the development of a cGMP-compliant process for manufacturing of human iPSCs (13) and suggested a comprehensive characterization approach (14) as an important step to develop high-quality iPSCs as input material. These iPSCs can be used at different manufacturing processes and, given their immortal status, can be utilized for many years or even decades. To demonstrate clinical relevance of these cells, we demonstrate here that our fully characterized human iPSC lines generated using cGMP-compliant process can readily differentiate into specialized cells from all three embryonic lineages with morphological and cellular characteristics of cardiomyocytes, definitive endoderm (DE), and neural stem cells (NSCs). Importantly, we also demonstrate how directed differentiation process can be further optimized to establish a robust and reproducible process as the main step in the development of a cGMP-compliant manufacturing possess to make clinical quantities of cell therapy products starting from the same iPSC lines.

## Materials and Methods

The human iPSC lines LiPSC-ER2.2 and LiPSC-18R were generated as described before (13) under cGMP-compliant environment and were continuously maintained in feeder-independent conditions using L7™ hPSCs Medium on defined L7 hPSC Matrix (Lonza, FP-5020). The L7 hPSC medium included L7 hPSC basal medium (Sartorious, 04-1191F) and L7 hPSC medium supplement (Sartorious, 04-1192J). The cells were serially passaged using L7 hPSC Passaging Solution (Sartorious, 04-1193E) per manufacturer’s protocol. All human iPSC lines were cultivated in a humidified 37°C cell incubator equilibrated with 5% CO2.

### Flow Cytometry

Flow cytometry of human iPSCs was performed when they reached around 70–80% confluency in human iPSC medium. The human iPSC cultures were then dissociated into a single-cell suspension using a TrypLE solution (ThermoFisher). The cells were fixed and permeabilized for intracellular staining with the 4% PFA and Perm/Wash buffer (Becton Dickinson), respectively. Permeabilized cells were incubated with Alexa-488 anti-OCT3/4 (Cell Signaling, 5177S) or respective Alexa-488 anti-IgG isotype control.

Extracellular antigens were detected on unfixed cells stained with PE-conjugated antigen-specific antibodies and respective isotypes using the manufacturer’s recommended concentration: anti-TRA-1-81 (Becton Dickinson, 560161), anti-TRA-1-60 (Becton Dickinson, 560193), anti-IgG3 isotype (Becton Dickinson, 556659), anti-SSEA4 (Becton Dickinson, 560128), and anti-IgM isotype (Becton Dickinson, 555584). The samples were then processed through a FACSCanto™ II flow cytometer (Becton Dickinson). Data were acquired using BD FACS Diva software and analyzed with Flowjo 7.6 software.

Flow cytometry of human iPSC-derived cells was performed after dissociation into single cells as follows: human iPSC-derived NSCs were washed once with PBS (-/-) and then dissociated into single cells using Accutase solution (Millipore, SCR005) for 6 min at 37°C as per manufacturer’s instruction. Same volume of neural induction medium supplemented with 10-µM Y-27632 (abcam, ab120129) was added to dilute the Accutase. Cell count and viability were determined and the cells were fixed and permeabilized for intracellular staining with 4% PFA and Perm/Wash buffer (Becton Dickinson, 554723), respectively. Permeabilized cells were incubated with Alexa Fluor 488 mouse anti-Pax6 (BD, PN561664) or respective Alexa Fluor 488 anti-IgG2a isotype control (BD, PN558055).

Human iPSC-derived Cardiomyocytes were washed once with PBS (-/-) and were treated with a mixture of Liberase (2.5 mg or 13 units/mL) and TrypLE solution (50:1) for 15 min at 37°C. The solution was neutralized with cardiac differentiation media (CDM). After 3–4 times pipetting, the cells were filtered using 100-µM cell strainer and were fixed and permeabilized for intracellular staining with the 4% PFA and Perm/Wash buffer, respectively. Permeabilized cells were incubated with anti-cTnT (abcam, ab8295) or respective anti-IgG isotype control (abcam, ab91353). The cells were washed and were incubated with PE goat anti-mouse IgG 2nd antibody (ThermoFisher, P21129). The samples were then processed through a FACSCanto™ II flow cytometer (Becton Dickinson). Data were acquired using BD FACS Diva software and analyzed with Flowjo 7.6 software.

### Immunocytochemistry

Human iPSC and iPSC-derived differentiated cells prepared for immunocytochemical analysis per following procedure: the culture medium was aspirated and washed twice with 1× Dulbecco’s Phosphate Buffered Saline (Lonza Biosciences, 17-513F). The cells were then fixed in 1× DPBS containing 4% PFA (Electron Microscopy Sciences, 15710) for 20 min, then permeabilized with 0.1% Triton X-100 (Sigma-Aldrich, T9284) in 1× DPBS for 15 min followed by a 15-min incubation with 10% goat serum in PBS in 0.1% Triton X-100 at room temperature. The cells were then treated with primary antibodies in 1% BSA in 1× DPBS overnight at 4°C. Primary antibodies against OCT4 (Abcam, ab19857; 1:350) and NANOG (R&D Systems, AF1997; 6.7 µg/mL), cTnT (abcam, ab8295, 1:200), Desmin (Dako, 1:300), Actin (Sigma, A111, 1:500), MYL2 (Sigma, HPA019763, 1:200), FoxA2 (abcam, ab108422, 1:300), Sox17 (R&D systems, MAB19241), Pax6 (Biolegend, 901301, 1:300), and Nestin (R&D systems, MAB1259, 1:50) were used in combination with the corresponding secondary antibodies Alexa Fluor 488 Goat anti-Rabbit (ThermoFisher, A11070, 1:1,000) and Alexa Fluor 594 Goat anti-mouse (ThermoFisher, A11005, 1:1,000). All cells were incubated with secondary antibodies and 5-µg/mL DAPI (ThermoFisher, D1306) in 1% BSA in 1× DPBS for 1 h at room temperature in the dark. Cells were rinsed between the incubation of the primary and secondary antibodies three times with 1% BSA in 1× DPBS. For the pluripotency surface marker staining, human PSCs [both iPSCs and embryonic stem cells (ESCs) if used as control] were treated with primary antibodies detecting extracellular antigens SSEA4 (Millipore, MAB4304; 1:100), TRA-1-60 (Millipore, MAB4360; 1:100), and TRA-1-81 (StemGent, 09-0011; 1:100) overnight at 4°C after the blocking step and prior to being permeabilized. All fluorescence detection was visualized using a Zeiss Observer.Z1 microscope equipped with ZEN software.

### Embryoid Body (EB) Differentiation

Confluent cultures of human PSC colonies were dissociated using L7 hPSC Passaging Solution. Cell aggregates were suspended in EB formation medium consisting of DMEM/F12 (Life Technologies, 11330-032) containing 10-µM Rock Inhibitor Y27632 (Millipore, SCM075) and allowed to settle down by gravity in a conical tube. After removing the supernatant, cells were suspended in fresh EB medium. Cell aggregates were then plated using a split ratio of 1:1 on Ultra Low Attachment (Corning, YO-01835-24) plates and returned to the incubator for 12 to 24 h. Once large cell aggregates formed, they were collected into a conical tube and allowed to settle by gravity. The medium was then replaced with differentiation medium (80% DMEM High Glucose) (Life Technologies, 11965-092), 20% defined fetal bovine serum (Hyclone, SH30070.03), 2-mM L-glutamine (Cellgro/Mediatech, 25-005-CI), 55-µM β-Mercaptoethanol (Life Technologies, 21985-023), and 1× non-essential amino acids (Life Technologies, 11140-050). The cell aggregates were placed on Ultra Low Attachment plates using a split ratio of 1:1 in 0.4 mL differentiation medium/cm^2^. The culture medium was then changed every other day for six days. On the seventh day, EBs were seeded on gelatin-coated plates [EmbryoMax^®^ ES Cell Qualified Gelatin Solution (Millipore, ES006-B)] at around 10 EBs/cm^2^. The EBs were allowed to attach undisturbed for 2 days. The differentiation medium was changed after the second day and every other day afterward with 0.4 mL/cm^2^ differentiation medium. The cultures were fixed and prepared for immunocytochemistry on day 14.

Spontaneously differentiated human PSCs were fixed with 4% PFA and permeabilized with 0.1% Triton X-100 PBS solution as described above. After rinsing the fixed cells with PBS-Tween solution, the cells were incubated with DPBS containing 10% goat serum (Life Technologies, 10000C) for 2 h at room temperature. Primary antibodies detecting alpha-1 Fetoprotein (Abcam, ab3980; 1:200 or R&D systems, MAB1369, 1:100), beta III tubulin (Millipore, MAB1637; 1:400), and Smooth Muscle Actin (DAKO, M0851; 1:500) were added to blocked cultures and incubated overnight at 2–8°C. The cells were rinsed twice with PBS-T, and the secondary antibody, Alexa Fluor 494-conjugated goat anti-mouse IgG(H + L) (Life Technologies, A-11032; 1:1,000) or Alexa Fluor 488-conjugated goat anti-mouse IgG(H + L) (Life Technologies, A11001; 1:1,000) were added and incubated for at least 2 h at room temperature. The cultures were then rinsed three times (5 min each) in 1× DPBS prior to counterstaining with DAPI. The cells were maintained in 50% glycerol for analysis.

### Neural Differentiation

Human iPSCs were maintained in Lonza L7 culture system. The cells were passaged using L7 passage solution to generate small clusters. Cells were plated with L7 medium onto L7 matrix-coated plates with 1:10 to 1: 20 ratio depending on the cell density. After 16–20 h, the medium was switched to Lonza Neural induction medium (3 mL/well of a 6-well plate, day 0) consisting of PNBM (Lonza, CC-3256), 1× B27 (ThermoFisher, 17504044), 1× Glutamax (ThermoFisher, 35050-061), 4-µM CHIR99021 (Tocris, 4423), 3-µM SB43152 (Stemgent, 04-0010), and 10-ng/mL hLIF (Millipore, LIF1010). The media were changed every other day until day 7. The cells were passaged using Accutase onto pre-coated wells. Y27632(5 µM/mL) was added to the neural induction medium. The cells were then plated at 1 million/well of 6-well plate which was pre-coated with Poly-L-Ornithine/Laminin (NSCs-P1). Next day, the medium was changed using neural induction medium (without Y27632). The medium was changed every other day until cells reach 95–100% confluency (4–5 days). The cells were passaged per above-mentioned procedure and placed on the wells pre-coated with Poly-l-Ornithine/Laminin (NSCs-P2). The cells were further expanded for flow cytometry and immunostaining (P3) and cryopreserved at P4.

### Coating Protocol for Culturing NSCs

Poly-l-Ornithine (Sigma, P4957) was diluted in water to final concentration of 20 µg/mL and was added to each well of a 6-well plate (1 mL/well). The plates were incubated at 4°C overnight or 37°C for 2 h. The wells were then rinsed twice with DPBS (without calcium/magnesium). Human-recombinant Laminin (Biolamina, LN521) was diluted in DPBS (with calcium/magnesium) to reach final concentration of 15 µg/mL. The Laminin solution was added to each well (1 mL/well) and incubated at 37°C at least for an hour but not longer than 6 h. The Laminin solution was aspirated just prior to plating the cells.

### Definitive Endoderm Differentiation

Human iPSCs were induced to differentiate into multipotent DE for 5 days using STEMdiff Definitive Endoderm kit (Stem Cell Technologies, 05110) per manufacturer’s protocol. Briefly, both iPSC lines were maintained at undifferentiated state for 3–4 passages using the L7 medium on L7 matrix prior to differentiation. The cells were washed with PBS on day 0, dissociated into single cell using TrypLE (ThermoFisher) and replated on L7 matrix-coated 6-well plates with 2.1 × 10^5^ cells/cm^2^ (i.e., 2 × 10^6^ cells/well) to reach around 100% confluency on day 1. The media were changed on day 1 to Medium 1 supplemented with factors A and B. Medium 2 supplemented with factor B was used for subsequent days. The cells were washed on day 5 with PBS, fixed with 4% PFA and stained for DE specific markers.

### Cardiomyocyte Differentiation

Human iPSCs were induced to differentiate into cardiomyocyte based on the protocol described by Burridge et al. with modifications (15). Human iPSCs were first maintained in undifferentiated state using Lonza L7 system or alternative for 2–3 passages prior to initiation of differentiation. The cells were around 95–100% confluency in 6-well plate on day 0. Importantly, human iPSCs were maintained at high-quality undifferentiated state with minimum spontaneous differentiation area before the start of CM induction (<5%). The media were changed to CDM consisting of RPMI1640 basal media (Lonza, 12-702F), 213-µg/mL L-ascorbic acid 2-phosphate (Sigma, A8960-5 mg) and 500 µg/mL O. sativa-derived recombinant human albumin (Sigma, A0237) supplemented with 6-µM CHIR99021 (Tocris, 4423). The cells were incubated at 37°C for two days (days 0–2) with another medium change on day 1. On day 2, the medium was changed to CDM supplemented with 2-µM Wnt-C59 (Tocris, 5148). The cells were incubated for another 2 days at 37°C with another medium change on day 3. The medium was changed on days 5 and 6 (3 mL/well) and every other day afterward until day 14 (2 mL/well) with CDM. First beating appeared around days 6–7 depending on the cell line. Representative images and videos were taken using Nikon Eclipse Ti inverted microscope using NIS Elements software. Cell counts and viability (CCV) measurements were performed using automated cell counter NucleoCounter NC-200 system (Chemometec, Denmark). Cardiomyocyte differentiation was performed with two cGMP lines LiPSC-18R and LiPSC-ER2.2 (*N* = 3).

### Alkaline Phosphatase Staining

Alpkaline phosphatase (AP) staining was performed using Millipore kit (cat. #SCR066) according to manufacturer’s protocol.

### Karyotype Analysis

Karyotype and STR analyses were performed by a qualified service provider (Cell Line Genetics, Inc., Madison, WI, USA) using standard G-banding methods as described elsewhere (14).

### Statistical Analysis

Cell count and viability data analysis were performed using GraphPad prism 6 software. Values are expressed as means (±SEM). Statistical comparisons were made using a one-way ANOVA, followed by multiple comparisons with a Tukey test, and a value of *P* < 0.0001 was considered statistically significant.

## Results

### Characterization of Human iPSCs Manufactured Using cGMP-Compliant Process

**Figure 1:**
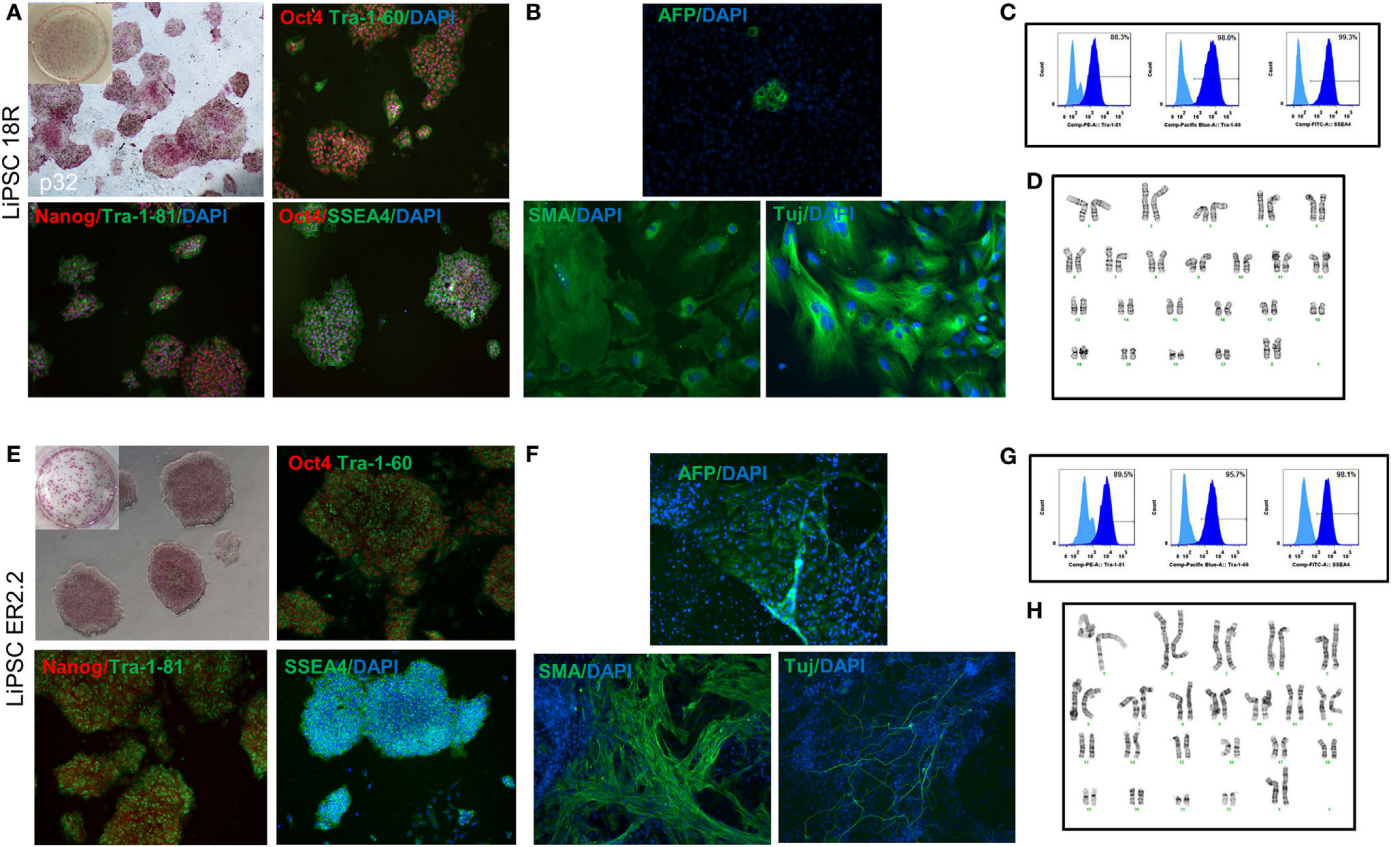
Characterization of current good manufacturing practice-compliant human-induced pluripotent stem cell (hiPSC) lines (LiPSC 18R and LiPSC ER2.2). **(A)** LiPSC 18R positively stained with pluripotency specific markers Oct4, Nanog, Tra-1-81, SSEA4, and Tra-1-60. hiPSCs were also positive for alkaline phosphatase (AP) (top left). **(B)** LiPSC 18R were induced to spontaneously differentiate into three germ layers through embryoid bodies (EB) formation. Differentiated cells expressed the markers for endoderm [Alpha-Feto Protein (AFP)], mesoderm [Smooth Muscle Actin (SMA)], and early ectoderm (beta-III-Tubulin or TUJ1). **(C)** Flow-cytometry analysis showed that the LiPSC 18R expressed the pluripotent stem cell surface markers including TRA-1-60, SSEA-4, and TRA-1-81 (dark blue). Light blue exhibits the isotype control. **(D)** LiPSC 18R demonstrated normal karyotype after eight passages in culture as shown by G-banding analysis. **(E)** LiPSC ER2.2 positively stained with pluripotency specific markers Oct4, Nanog, Tra-1-81, SSEA4, and Tra-1-60. hiPSCs were also positive for alkaline phosphatase (AP) (top left). **(F)** LiPSC ER2.2 were induced to spontaneously differentiate into three germ layers through EB formation. Differentiated cells expressed the markers for endoderm (AFP), mesoderm (SMA), and early ectoderm (beta-III-Tubulin or TUJ1). **(G)** Flow-cytometry analysis showed that the LiPSC ER2.2 expressed the pluripotent stem cell surface markers including TRA-1-60, SSEA-4, and TRA-1-81 (dark blue). **(H)** LiPSC 18R demonstrated normal karyotype after 15 passages in culture as shown by G-banding analysis.

Two human iPSC lines were, LiPSCs-18R and LiPSC-ER2.2, generated using cord blood CD34 + cells and a cGMP manufacturing process reported elsewhere (13). To ensure the quality of starting iPSCs, the cells were thawed in L7™ hPSC medium and L7™ Matrix, serially subcultured using L7™ hPSC passaging solution and underwent standard characterization and safety studies. Evaluation of the iPSC colonies was initially performed based on their morphology after thaw, staining for AP, PSC markers, and spontaneous differentiation. Both lines exhibited positive AP colonies and stained positive for Oct4, Nanog, SSEA4, Tra-1-81, and Tra-1-60 (Figures 1A,E) and exhibited differentiation into three embryonic germ layers (ectoderm, mesoderm, and endoderm) through embryoid body (EB) formation (Figures 1B,F). Flow-cytometry analysis also confirmed high percentage of pluripotency expression in both lines (Figures 1C,G). Karyotype analysis of both human iPSC lines showed normal female karyotype (46, XX) without any apparent chromosomal abnormalities (Figures 1D,H).

### Differentiation of cGMP-Compliant Human iPSCs Into Cardiac Lineage Under Defined Conditions

**Figure 2:**
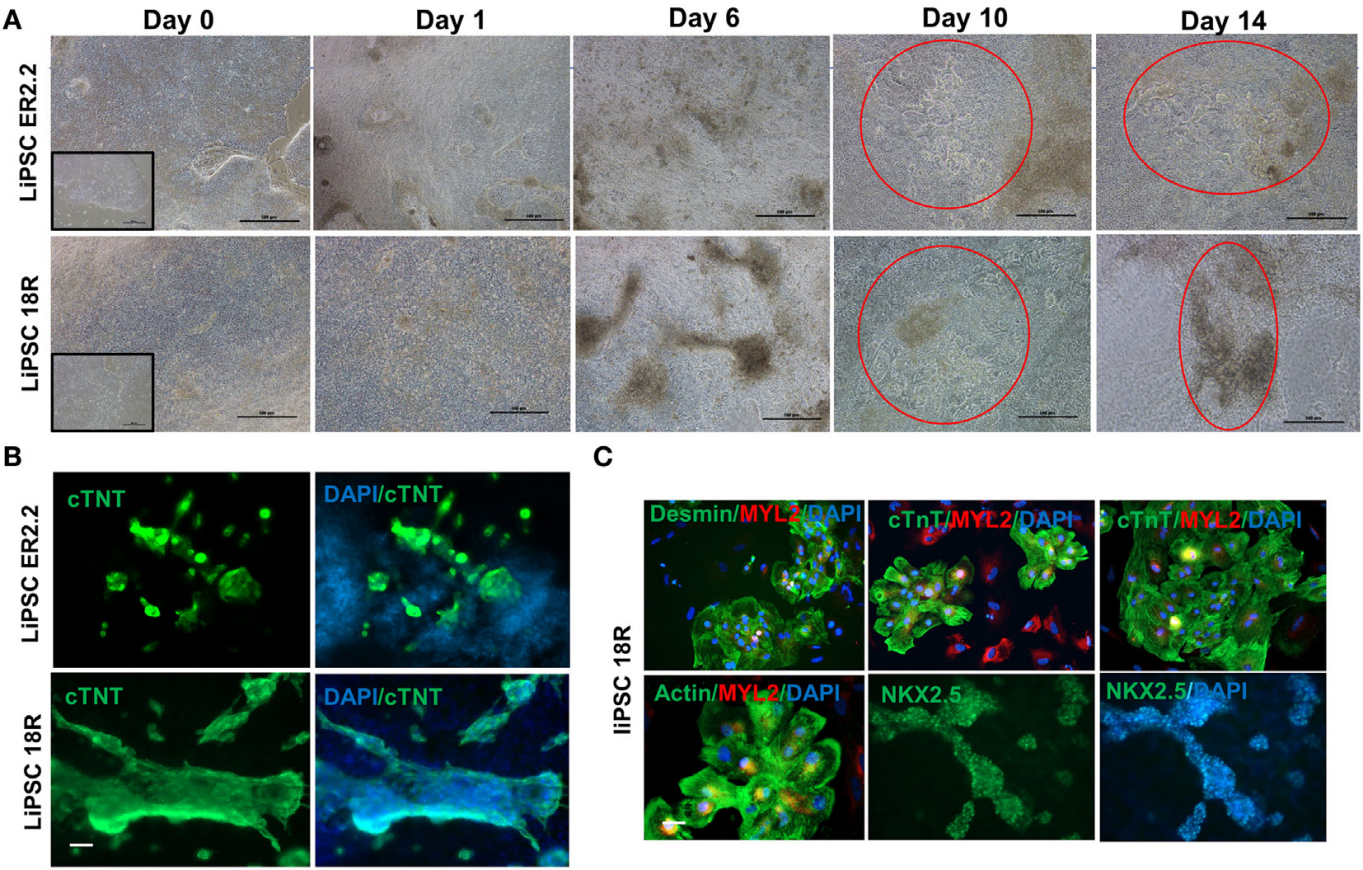
Both current good manufacturing practice-compliant LiPSCs lines (18R and ER2.2) differentiated into beating cardiomyocytes in two-dimensional (2D) culture system. **(A)** starting with good quality cells (around 100% confluency with very low differentiated areas), both hiPSC lines started morphological changes from day 1 onward until day 14. The first beating observed for both lines around days 6–7 within several patches. A large beating area has been shown for each line on days 10 and 14 (red circles). **(B)** All of the beating areas on day 14 of differentiation were positively stained for cardiac-specific troponin (cTnT) marker for both hiPSC lines. A representative large cardiac patch (a few millimeter size) has been shown here for each line. **(C)** LiPSC 18R was characterized for more cardiomyocyte markers (Desmin, myosin light chain 2, and Actin) and stained positive for cardiac progenitor marker (NKX2.5). Scale bar: 100 µm.

Using a defined culture condition based on the established protocols (15), human iPSCs were differentiated into cardiomyocytes for 14 days in 2D cell culture system on L7 matrix. Three different PSCs culture systems were initially evaluated prior to the initiation of the CM differentiation including TeSR-E8 (Stem Cell Technologies), Nutristem (Biological Industries), and L7 cell culture system. Human iPSCs grown in L7 hPSC medium and Nutristem could develop and differentiate into patches of beating CMs. However, TeSR-E8 failed to support PSCs differentiation due to the cell loss and lack of proliferation between days 1 and 6. No beating area was observed in the cells maintained in TeSR-E8 system (Figures S1 and S2 in Supplementary Material). Human iPSCs grown in L7 and NutriStem systems generated more than 39 and 36% of cTnT positive cells on day 10 post-differentiation, respectively (Figure S3A in Supplementary Material). They also developed patches of contracting cells as shown by immunofluorescent staining (Figure S3B in Supplementary Material). We further evaluated the cardiomyocyte differentiation using the cells grown in L7 hPSC medium. During the differentiation state, the morphology of the cells rapidly changed during the wnt pathway induction and inhibition (days 0–4) and continued until day 14 (Figure 2A). For both iPSC lines, the first beating area appeared on day 7 or 8 post-induction. The beating cells were initially confined to a small area but expanded gradually to various sizes (ranging from 500 µm to a few millimeter) of synchronized beating areas (Figure 2A; Videos S1–S4 in Supplementary Material). Immunofluorescent staining of the differentiated cells on day 14 showed expression of cardiomyocyte specific markers including cTnT, Nkx2.5, MYL2, Desmin, and Actin (Figures 2B,C). Although not quantified, the majority (>90%) of cells in a beating cardiac area were stained positive for the above markers on day 14. Viability of harvested cells on day 14 was >82% in all runs and each well of a 6-well plate generated more than 2.5 × 10^6^ viable cells, although there was some variability between runs (Figure S4 in Supplementary Material). To show the persistence of beating cells *in vitro*, the cells were maintained in the CDM for longer period without passaging. The patches of cardiomyocytes continue to contractile for 5 weeks.

### Development of a Robust and Reproducible Cardiac Differentiation Process

**Figure 3:**
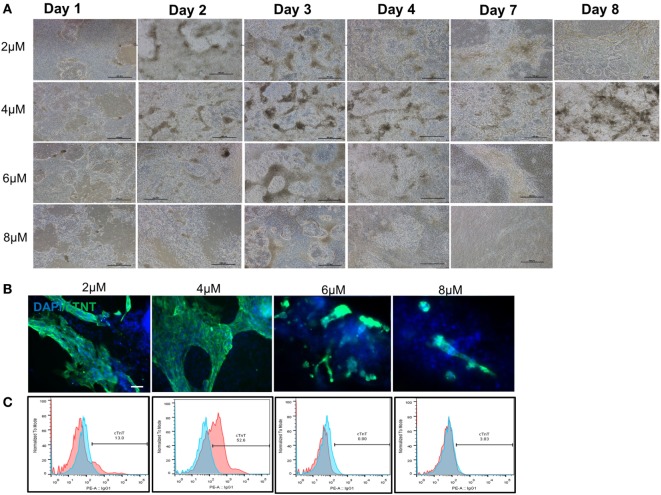
Optimization of cardiomyocyte differentiation through modulation of CHIR99021 concentration. **(A)** Morphology of LiPSC 18R-derived differentiated cells was changed enormously during the first week of directed differentiation. The cells with 8 µM of CHIR resulted in lots of cell death and empty spaces with no beating area observed. Dark brownish areas are representative of cardiac beating patches. **(B)** Immunofluorescent staining of differentiated cells for cardiomyocyte specific marker cardiac-specific troponin (cTnT) demonstrated that 4 µM of CHIR99021 resulted in higher number and bigger beating areas among the four conditions tested. **(C)** Flow cytometry for cTnT also confirmed the higher number of cardiomyocytes in the 4-µM condition (pink: target, blue: isotype). Scale bar: 100 µm.

After demonstrating the feasibility of inducing cGMP-compliant iPSCs into cardiac lineage, a gap assessment study revealed a few gaps in the differentiation process that led to inefficient and suboptimal cardiac differentiation. Aside from evaluation of the cell culture condition at iPSC stage (as described earlier), initial plating density, CHIR concentration, and washing cells during the differentiation process were among the most important factors studied during process optimization. Our results showed that washing the cells, especially during differentiation period, resulted in larger area of beating patches of cardiomyocytes (data not shown). The density of human iPSCs on day 0 also showed relatively high impact on subsequent differentiation. The cells had to reach to around 100% confluency prior to differentiation to prevent cell loss during the first 4 days and successful process. We also evaluated optimal CHIR component concentrations at 2.0, 4.0, 6.0, and 8.0 µM. Our results demonstrated that 4.0-µM CHIR99021 resulted in a highest number of cell survival during day 1 to day 4 and the highest number of cardiac beating areas (Figure 3A). The cell loss was higher in the 6- and 8-µM concentrations considering the absence and/or detachment of cells on days 1 and 2. Immuno-staining for cTnT confirmed that 4 µM of CHIR99021 produced large patches of cardiomyocytes (Figure 3B). In some of the wells, the entire area was beating starting from day 10 afterward until day 14 (Videos S5 and S6 in Supplementary Material). Flow cytometry of day 14 cells further confirmed the higher effectiveness of 4-µM CHIR99021 on the CM directed differentiation of LiPSC 18R (around 53% TnT), while 2 µM resulted in around 13% TnT expressing cells during followed by 6 and 8 µM which yielded the lowest TnT (Figure 3C).

### Differentiation of cGMP-Compliant Human iPSCs into Ectoderm and Endoderm Lineages

**Figure 4:**
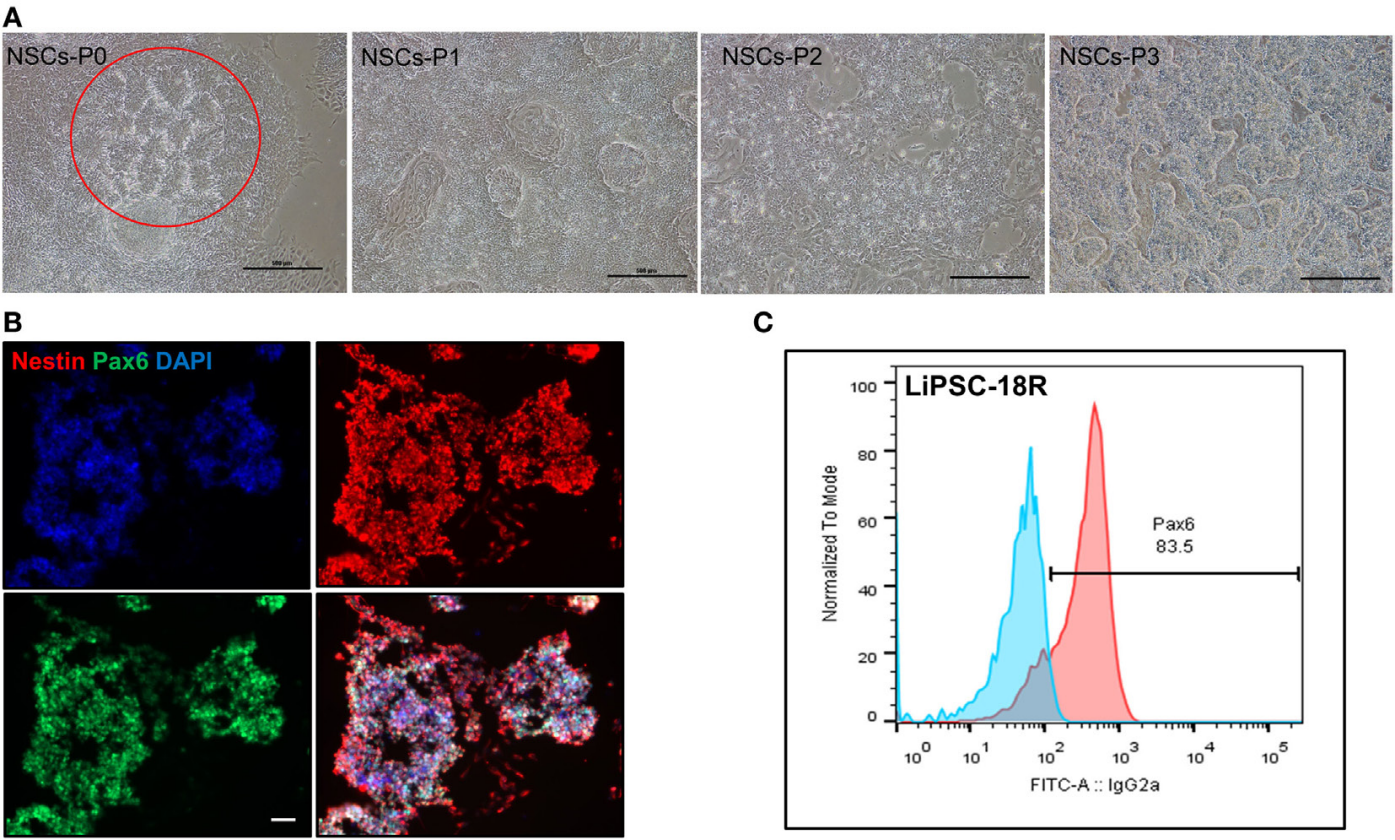
Differentiation of LiPSCs into neural stem cells (NSCs). **(A)** Morphology of LiPSC 18R-derived cells was changing during the differentiation from P0 to P3. Red circle shows the neural rosette-like structure at the end of P0. **(B)** Immunofluorescent staining of NSCs at the end of P3 revealed that they express NSC-specific markers (Nestin and Pax6). **(C)** Flow-cytometry analysis at the end of P3 demonstrated that around 83.5% of cells acquired NSC morphology. Scale bar: 100 µm.

In order to demonstrate the potential of directed differentiation of LiPSCs-18R and LiPSC-ER2.2, the iPSCs were induced into NSCs (ectoderm) using a protocol originally established by Li et al. (16). Treatment of LiPSCs with hLIF and 2 small molecules (CHIR99021 and SB431542) induced the cells into a primitive NSCs by modulation of canonical Wnt signaling (GSK3 inhibition) (17) and TGF-beta/Activin receptors (18). Some key modifications and optimizations were included in the protocol including replacement of feeder with feeder-free culture system, plating density during differentiation, ROCK inhibitor treatment time, and the basal media (data not shown). Prior to the differentiation, human iPSCs were thawed in L7™ hPSC medium and L7™ Matrix and serially subcultured using L7™ hPSC Passaging Solution. Differentiation to NSCs was monitored by the appearance of morphology changes and epithelial/rosette-like structures formation at the end of day 7 and during the entire process (Figure 4A). These cells were expanded for 24 days (three passages) and cryopreserved at the end of each passage. Immunofluorescent staining for Pax6 (an early marker of neural induction) and Nestin indicated the acquisition of NSCs phenotype by the majority of LiPSCs 18R at the end of passage 3 (Figure 4B). Consistent with this observation, flow-cytometry analysis showed that more than 80.0% of cells expressed Pax6 on day 24 (P3) (Figure 4C). These homogenously differentiated neural cells could be stably expanded on Poly-L-Ornithine and Laminin in the presence of hLIF, CHIR99021, and SB431542.

**Figure 5:**
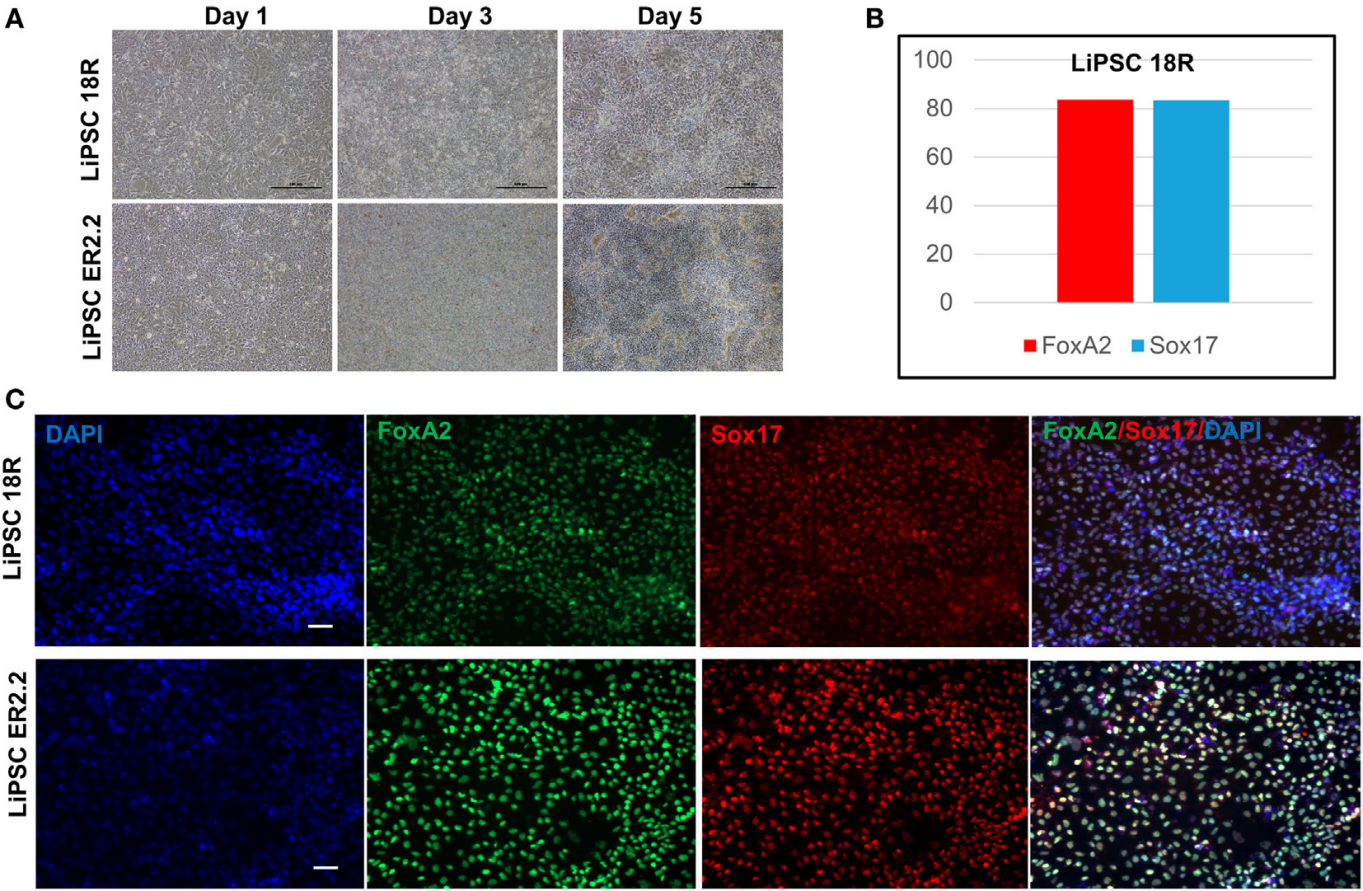
Differentiation of LiPSCs into definitive endoderm (DE). **(A)** Morphology of LiPSC-derived cells was changing during the differentiation from day 1 to day 5. The cells were highly proliferative during the differentiated period and produced dense single-layered sheets. **(B)** Flow-cytometry analysis at the end of day 5 demonstrated that more than 80% of LiPSC 18R-derived cells express FoxA2 and Sox17. **(C)** Immunofluorescent staining of day 5 cells on both lines revealed that they express DE-specific markers (FoxA2 and Sox17). Scale bar: 100 µm.

Human iPSCs were induced to DE lineage using a commercially available kit. High Activin A (100 ng/mL) is generally used to induce human ESCs and iPSC differentiation to DE (19, 20), while lower concentrations induce mesodermal lineage differentiation (21, 22). Activin A is a member of transforming growth factor beta (TGF-β) family of proteins produced by many cell types throughout development and has an important impact on the variety of cell functions including proliferation, apoptosis, and metabolism (23). Human iPSCs started morphological changes on day 1 until day 5 (Figure 5A). Flow cytometry demonstrated that more than 80% of LiPSC-18R-derived cells were positive for Sox17 and FoxA2 as specific markers for DE (24) on day 5 (Figure 5B). Differentiation to DE was further confirmed by the acquisition of endoderm marker expression (FOXA2 and SOX17) by both human iPSC lines using immunofluorescent staining technique (Figure 5C).

## Discussion

We have previously reported the development of a cGMP manufacturing process to generate clinically compliant human iPSC lines and detailed characterization of these cells (13, 14). These studies have addressed some of the challenges associated with manufacturing of high-quality iPSCs under cGMP-compliant conditions, including tissue sourcing, manufacturing, testing, and storage. Using a cGMP-compliant human iPSC manufacturing process, a feeder- and xeno-free derivation and cell culture system as well as well characterized starting cells are important prerequisites toward development of clinically relevant differentiation products. Here, we used two of our cGMP-compliant iPSC lines and evaluated their potential to generate specialized cells from three lineages. Although cGMP-compliant cells have been already generated from human ESCs, most of the cells were qualified for cGMP by additional testing after derivation under non-cGMP environment (25, 26). Recently, clinically grade patient-specific iPSCs and photoreceptor precursor cells were produced under xeno-free and cGMP-compliant in an academic facility (27). There are still major obstacles toward clinical application of human iPSC: high cost of production and post-transplantation immunological reaction, which highlights the need for generation of large HLA-matched MCBs from donors with selected haplotypes (28, 29) or by genetic manipulation of human iPSC prior to differentiation (28) to make them suitable for allogenic transplantation.

Two human iPSC lines used in this study were generated using the cGMP iPSC manufacturing process by applying best practices for cell culture, documentation, and quality control (30) as described in our previous report (13, 14). The quality of these iPSCs was comprehensively evaluated based on the number of alkaline phosphatase positive iPSC colonies, morphology and expression of pluripotency markers using flow cytometry and immunostaining. In order to demonstrate the clinical potential of iPSCs for allogenic transplantation, the cells need to exhibit their potential to differentiate into cells from three embryonic lineages in unbiased manner.

Our 2D induction strategy to differentiate human iPSC into cardiomyocyte was based on a published procedure (15), and we further optimized the process to use with our defined L7 culture system by evaluating initial cell density, confluency of cells on day 0, and the effect of washing step on subsequent CM differentiation. Starting with the confluency of near 100% and adding a washing step during media feed, the total spontaneous cardiac beating areas, total cardiomyocyte yield and cell viability increased at day 14 and cell loss decreased significantly especially during the first 6 days. The effect of human iPSC split ratio and starting cell density on differentiation into various lineages have been reported in other studies (31, 32). Additionally, concentration of cytokines/small molecules for the Wnt signaling modulation is highly dependent upon the cell confluency. During optimization, the fibroblast-like cells and cystic structures appeared at minimum number in the culture with 4-µM CHIR99021 compared with other conditions. This highlights the effect of small molecule concentration on the cell fate and yield. Recently, a sequential multi-layer 2D culture system for the expansion of human iPSC and generation of iPSC-derived CMs was reported (33). This study highlighted that upon optimizing CM differentiation the 2D system can generate a large number of human iPSC-derived CMs with high efficiency for clinical applications. Recent studies demonstrated that human iPSCs can generate various types of cardiomyocytes depending on the elapsed time in culture (Ventricular, Atrial, or Nodal) and depletion of the medium from Glucose and Glutamine (15, 33). Although the CM differentiation was optimized, we did not evaluate the subtype of CMs, but according to published reports (15), the majority of CMs may be atrial subtype. Moreover, our human iPSC-CMs exhibited a fetal phenotype based on their proliferation status and time in culture. Although CMs gradually lost their proliferation capacity, they continued to beat spontaneously for 35 days in culture on L7 matrix without subculturing. Furthermore, we believe that the induction efficiency and the type of CMs are not only dependent on the specific iPSC line but also their method of reprogramming. This factor needs to be independently determined. Recently, we have been able to adapt and modify the 2D CM differentiation protocols to differentiated human iPSCs into CM in 3D suspension culture (manuscript under preparation). Using 3D directed differentiation, it would be feasible to generate high-quantity CM under computer controlled conditions.

We also examined differentiation capability of cGMP-compliant iPSCs into neural and endodermal lineages by differentiation into NSCs and definitive endoderm, respectively. Differentiation of PSCs into NSCs and functional neurons has been reported by several groups using different cytokines and small molecules (16, 34, 35). Furthermore, several groups have established methods to derive and characterize functional dopaminergic and motor neurons from PSCs of normal and affected individuals (8, 36–41). Recently, dopaminergic neurons derived from hESCs and manufactured by a cGMP-compatible process could engraft and survive in a rat model of Parkinson’s disease (PD) (42). To the best of our knowledge, no cGMP-compliant PSC has been induced to acquire NSCs phenotype. Using the NSCs differentiation procedure published in the literature (16), we first evaluated feasibility of differentiation into NSCs and then optimized the process to improve differentiation efficiency and reproducibility for cGMP-compliant human iPSC lines. We replaced the inactivated mouse embryonic fibroblasts (MEFs)- and matrigel-based culture system with L7 culture system to have a completely defined, serum and xeno-free media. This is a major step in development of a cGMP-compliant bioprocess to avoid lot-to-lot variability, undefined, and potentially compromised culture condition. The cells were induced into differentiation on a feeder-independent (L7 matrix) system using Lonza PNBM media and optimized CHIR99021 and SB43152 concentrations. Moreover, a defined substrate (Poly-L-Ornithin and recombinant Laminin) was used during the differentiation stage to avoid use of animal-derived components in the cell culture system. Implementing these modifications, it was possible to achieve a high yield of epithelial-like NSCs as exhibited by high expression of Pax6 and Nestin. The cells were highly proliferative especially during the first three passages, which showed their self-renewal potential. We have recently reported advantages of using L7 hPSC culture system for the expansion of PSCs and their differentiation potential using spontaneous differentiation method (EB formation) (43). Here, we demonstrate that directed differentiation protocols can be used for human iPSCs expanded using the defined L7 cell culture system to efficiently differentiate into specialized cells of all three germ layers. The main reason for successful controlled differentiation of these iPSCs lies in the fact that L7 PSCs culture media (both basal medium and growth supplements) have been specifically designed to include optimum level of medium components and does not include very high level of growth factors or cytokines (e.g., bFGF) compared with other commercially available PSCs media. This formulation supports expansion and maintenance of high-quality PSCs (both iPSCs and hESCs) at undifferentiated state, but most importantly primes the cells to quickly respond to environmental cues with lower concentration of cytokines/small molecules in a shorter time span. This is an important consideration for clinical application of PSCs where high quality, safety, low cost (which mainly results from the differentiation and manufacturing time) are essential factors in the manufacturing of terminally differentiated cells. In addition, we have shown that using optimized culture conditions it is possible to retain the ability of iPSCs to differentiate into three lineages when starting from the same cGMP-compliant starting material and that separate MCB banks may not be required. This can be a huge cost saving strategy in development of high-quality iPSC-derived products.

Recent studies have demonstrated the influence of the epigenetic memory, donor cell source, state of pluripotency (naïve vs. primed) and number of reprogramming factors on the lineage differentiation and characteristics of terminally differentiated cells (44–49). Specifically, in one study, Sanchez-Freire et al. reported the effect of cell source and epigenetic memory on cardiomyocyte differentiation of human iPSCs which was shown to be higher in cardiac progenitor cells compared with skin fibroblasts (50). This phenomenon could be resolved with extended passaging and time in culture to erase the epigenetic memory (50, 51). Additionally, epigenetic memory and cell line origin might make some human iPSC lines more refractory or amiable to external differentiation stimulations into certain lineages which should be evaluated experimentally for each line prior to manufacturing MCB for specific clinical applications.

In summary, here we report a viable proof-of-concept strategy to directly differentiate the same cGMP-compliant iPSC lines into the specialized cells from three embryonic lineages. Considering their cGMP compliance, comprehensive characterization and unbiased differentiation to all three lineages and manufacturing readiness, we believe that these human iPSCs hold the potentials to generate clinically relevant products for future clinical applications.

## Author Contributions

MS wrote the manuscript, designed process development and optimization studies, and performed the experiments. FY designed process development and performed experiments. TF and MR provided intellectual support. BB wrote the manuscript, designed process development and optimization studies, and provided supervision and intellectual guidance. All authors have read and approved the final version of the manuscript.

## Conflict of Interest Statement

The authors declare that the research was conducted in the absence of any commercial or financial relationships that could be construed as a potential conflict of interest. The reviewer DH and the handling editor declared their shared affiliation.

## Supplementary Material

Figure S1 Differentiation of human-induced pluripotent stem cells (iPSCs) maintained in three commercially available culture systems. Human iPSCs were expanded and maintained undifferentiated in 2D system using Nutristem, TeSR-E8 and L7 media cultured on Laminin, Vitronectin, and L7 matrix, respectively. Cells reached around 90–95% confluency on day 0 prior to cardiomyocyte differentiation. The cells maintained on TeSR-E8 showed the highest cell death during the first three days. Scale bar: 500 µm.

Figure S2 Beating areas were observed around day 7. Patches of beating areas were only seen in the Nutristem and L7 culture systems, while no beating was observed in the human-induced pluripotent stem cells maintained in TeSR-E8. TeSR-E8 showed the highest cell loss. Scale bar: 500 µm.

Figure S3 Characterization of human-induced pluripotent stem cell-derived cardiomyocytes. **(A)** Flow-cytometry analysis demonstrated that the cells differentiated from Nutristem and L7-maintained cells showed around 40 and 37% cardiac troponin (cTnT) expression. **(B)** IF staining showed patches of cTnT-positive cardiomyocytes in Nutristem and L7-maintained cells. Scale bar: 100 µm.

Figure S4 Comparison of cell number and viability after harvest. Human-umHHinduced pluripotent stem cells were harvested on day 14 of cardiomyocyte differentiation using Liberase/TrypLE enzyme mix. Cell count and viability was measured. **(A)** The viable cell yield from one well of a 6-well plate was between 2.5 and 3.5 × 10^6^ cells. **(B)** The viability of over 82% was achieved in all three runs. n.s: not significant.

Video S1 LiPSC ER2.2-derived cardiomyocytes beatings on day 8 post differentiation.

Video S2 LiPSC ER2.2-derived cardiomyocytes beatings on day 14 post differentiation.

Video S3 LiPSC 18R-derived cardiomyocytes beatings on day 11 post differentiation.

Video S4 LiPSC 18R-derived cardiomyocytes beatings on day 14 post differentiation.

Video S5 LiPSC 18R-derived cardiomyocytes beatings on day 14 post differentiation (2 µM of CHIR99021).

Video S6 LiPSC 18R-derived cardiomyocytes beatings on day 14 post differentiation (4 µM of CHIR99021).
