# Supplementary figures and images for: Human-Induced Pluripotent Stem Cells Manufactured Using a Current Good Manufacturing Practice-Compliant Process Differentiate Into Clinically Relevant Cells From Three Germ Layers
Source: Front Med (Lausanne). 2018 Mar 15;5:69. doi: 10.3389/fmed.2018.00069 (PMC5862873; doi:10.3389/fmed.2018.00069)

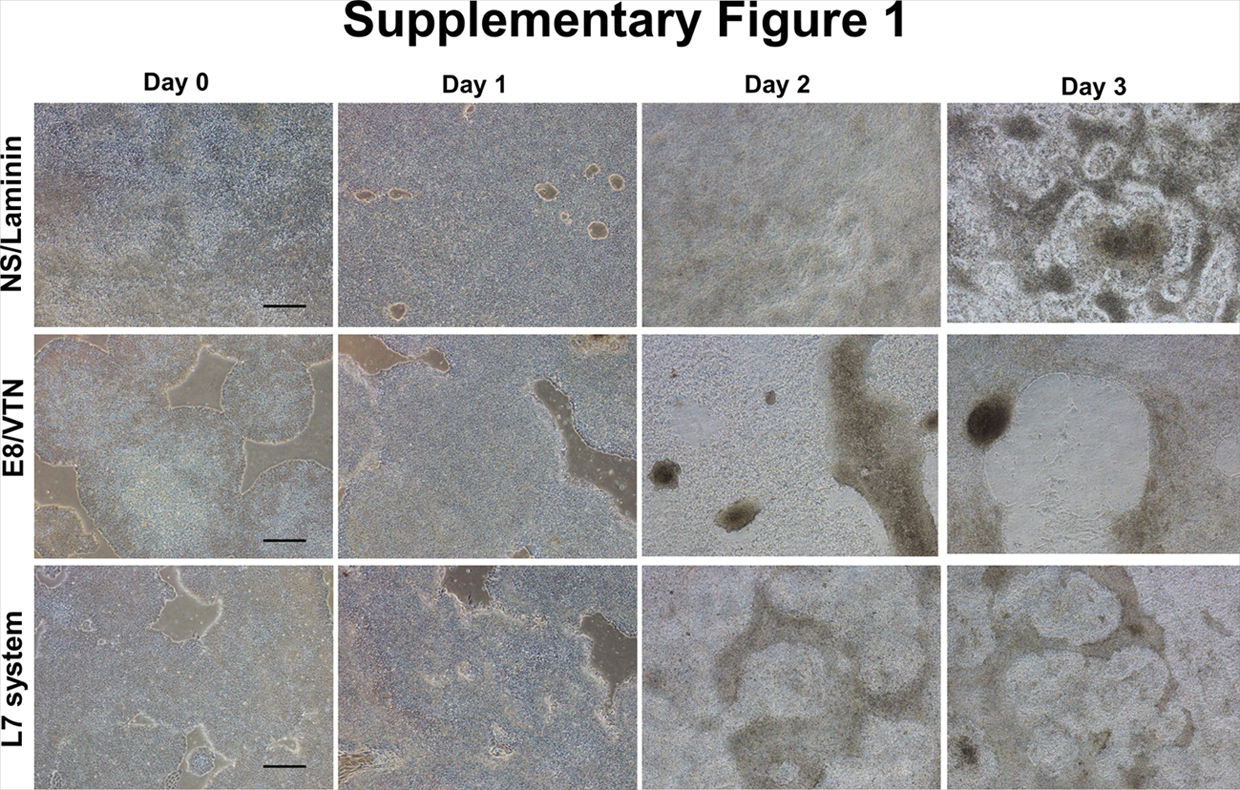

Supplement: Figure S1 — Differentiation of human-induced pluripotent stem cells (iPSCs) maintained in three commercially available culture systems. Human iPSCs were expanded and maintained undifferentiated in 2D system using Nutristem, TeSR-E8 and L7 media cultured on Laminin, Vitronectin, and L7 matrix, respectively. Cells reached around 90–95% confluency on day 0 prior to cardiomyocyte differentiation. The cells maintained on TeSR-E8 showed the highest cell death during the first three days. Scale bar: 500 µm. [file image_1.tif]

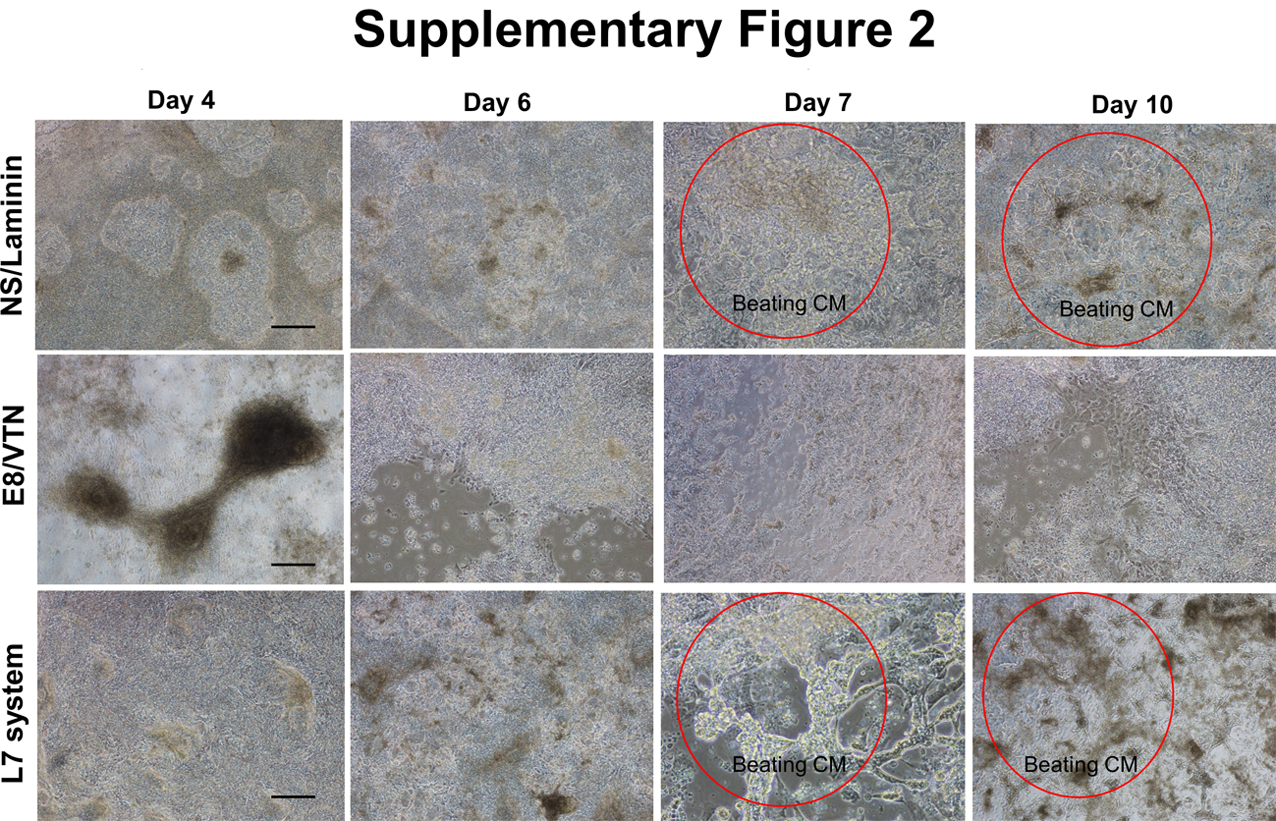

Supplement: Figure S2 — Beating areas were observed around day 7. Patches of beating areas were only seen in the Nutristem and L7 culture systems, while no beating was observed in the human-induced pluripotent stem cells maintained in TeSR-E8. TeSR-E8 showed the highest cell loss. Scale bar: 500 µm. [file image_2.tif]

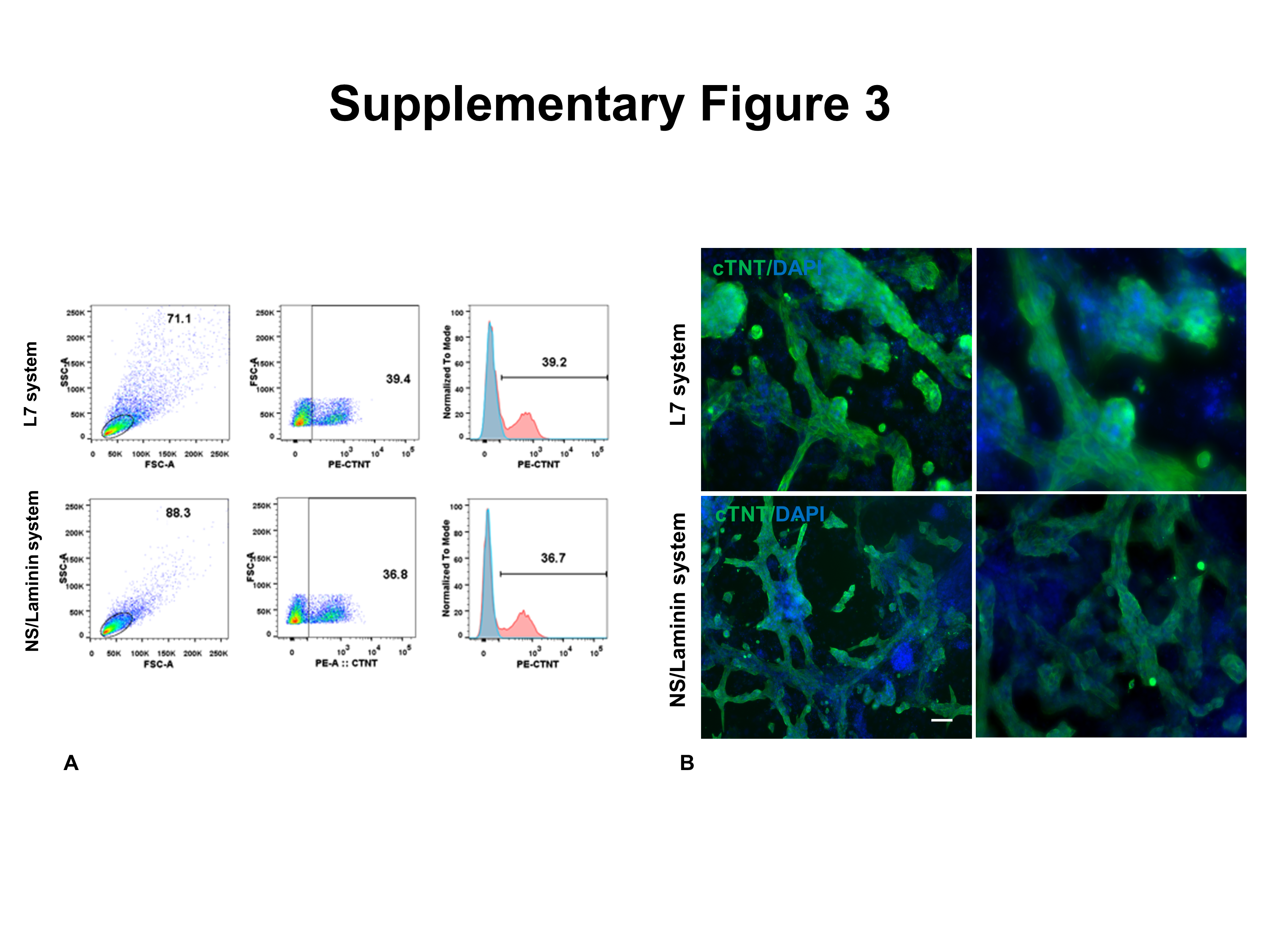

Supplement: Figure S3 — Characterization of human-induced pluripotent stem cell-derived cardiomyocytes. (A) Flow-cytometry analysis demonstrated that the cells differentiated from Nutristem and L7-maintained cells showed around 40 and 37% cardiac troponin (cTnT) expression. (B) IF staining showed patches of cTnT-positive cardiomyocytes in Nutristem and L7-maintained cells. Scale bar: 100 µm. [file image_3.tif]

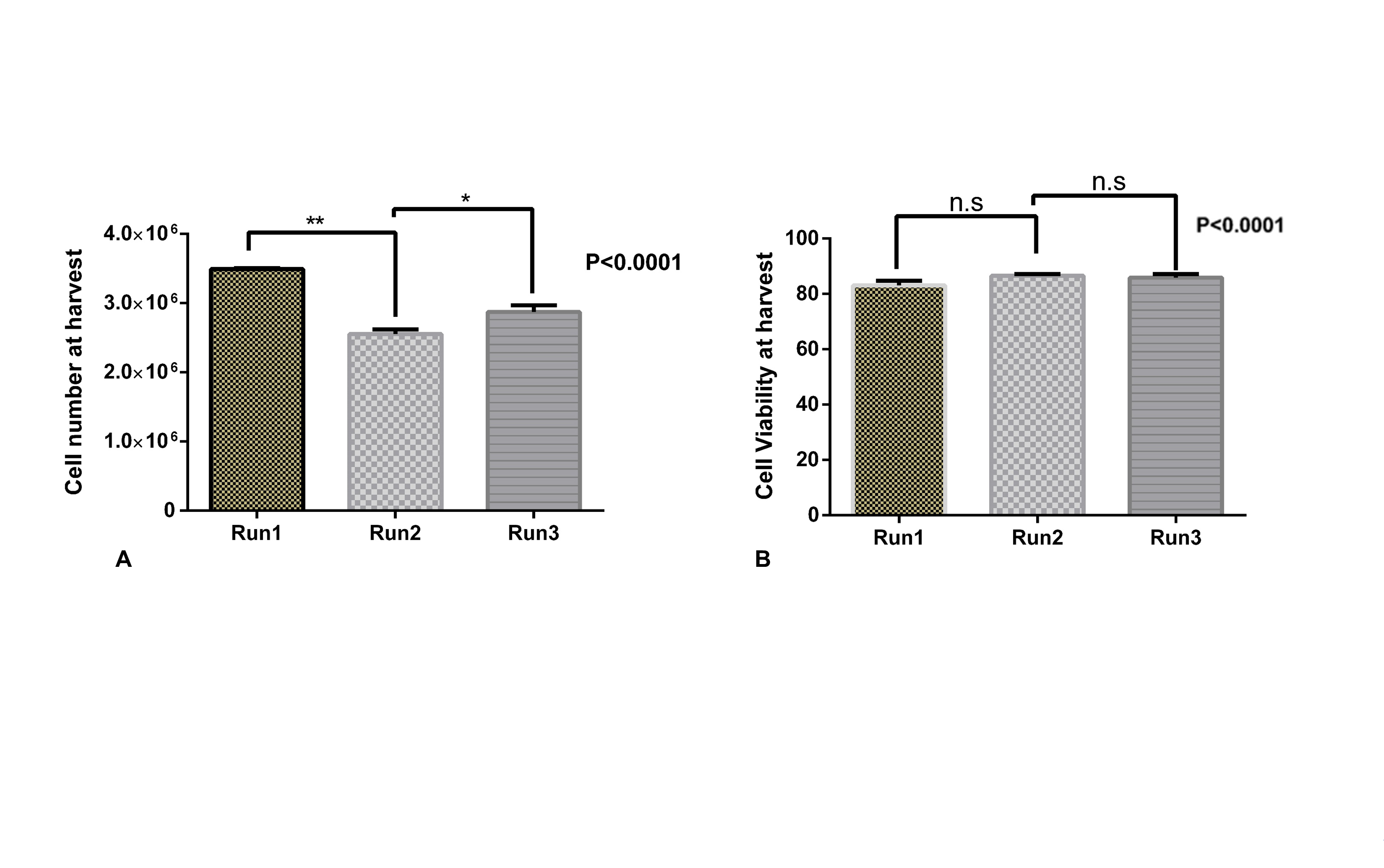

Supplement: Figure S4 — Comparison of cell number and viability after harvest. Human-umHHinduced pluripotent stem cells were harvested on day 14 of cardiomyocyte differentiation using Liberase/TrypLE enzyme mix. Cell count and viability was measured. (A) The viable cell yield from one well of a 6-well plate was between 2.5 and 3.5 × 10^6^ cells. (B) The viability of over 82% was achieved in all three runs. n.s: not significant. [file image_4.tif]
